# Reference Values for Inspiratory Muscle Endurance in Healthy Children and Adolescents

**DOI:** 10.1371/journal.pone.0170696

**Published:** 2017-01-25

**Authors:** Cristhiele Taís Woszezenki, João Paulo Heinzmann-Filho, Fernanda Maria Vendrusculo, Taila Cristina Piva, Isadora Levices, Márcio Vinícius Fagundes Donadio

**Affiliations:** 1 Graduate Program in Pediatrics and Child Health, Pontifícia Universidade Católica do Rio Grande do Sul, Porto Alegre, Rio Grande do Sul, Brasil; 2 Laboratory of Pediatric Physical Activity, Centro Infant, Institute of Biomedical Research, Pontifícia Universidade Católica do Rio Grande do Sul, Porto Alegre, Rio Grande do Sul, Brasil; 3 School of Nursing, Nutrition and Physiotherapy, Pontifícia Universidade Católica do Rio Grande do Sul, Porto Alegre, Rio Grande do Sul, Brasil; West Virginia University School of Medicine, UNITED STATES

## Abstract

**Aims:**

To generate reference values for two inspiratory muscle endurance (IME) protocols in healthy children and adolescents.

**Materials and methods:**

This is an observational, cross-sectional study, in healthy children and adolescents from 4 to 18 years of age. Weight, height, maximal inspiratory pressure (MIP) and IME were measured using two protocols. A fixed load of 30% of MIP with a 10% increment every 2 minutes was used in the incremental threshold loading protocol. As for the maximal loading protocol, a fixed load of 70% of MIP was used and the time limit (Tlim) achieved until fatigue was measured.

**Results:**

A total of 462 participants were included, 281 corresponding to the incremental loading protocol and 181 to maximal loading. There were moderate and positive correlations between IME and age, MIP, weight and height in the incremental threshold loading. However, the regression model demonstrated that MIP and age were the best variables to predict the IME. Otherwise, weak and positive correlations with age, weight and height were found in the maximal loading. Only age and height influenced endurance in the regression model. The predictive power (r^2^) of the incremental threshold loading protocol was 0.65, while the maximal loading was 0.15. The reproducibility measured by the intraclass correlation coefficient (ICC) was higher in the incremental loading (0.96) compared to the maximal loading test (0.69).

**Conclusion:**

IME in healthy children and adolescents can be explained by age, height and MIP. The incremental threshold loading protocol showed more reliable results and should be the model of choice to evaluate IME in the pediatric age group.

## Introduction

Inspiratory muscle endurance (IME) can be defined as the capacity of a muscle or a muscle group to sustain a particular task over time, being directly related to muscle fatigue [1, 2]. The evaluation of IME is a simple, non-invasive and easy to apply method, which can be measured through sustained maximal voluntary ventilation, incremental threshold loading test or maximal loading test [3].

The incremental threshold loading test is a method that initially uses low resistance loads, giving the individual a learning period during the initial steps, increasing the output load progressively until exhaustion [4]. As for the maximal loading, a fixed load of the maximal inspiratory pressure (MIP) is used throughout the test. The final result is defined as the time limit (Tlim) achieved until exhaustion [3]. Previous studies have demonstrated that both protocols are frequently used in the adult age group, even though some findings indicate that the maximal loading protocol presents a higher variability in the IME measurement when compared to the incremental threshold loading protocol [5, 6].

In clinical practice, the IME evaluation is used to determine muscle fatigue, quantify the severity of certain diseases and also for its prognostic value [7]. In children, IME can assist in the management and follow up of pulmonary diseases, for instance, asthma and cystic fibrosis, and it is used in rehabilitation programs [8]. Moreover, the comparison or interpretation of the results of the IME tests is limited by the fact that there are no normal or reference values for the pediatric population.

In this context, it is relevant to obtain reference values that could help to better quantify respiratory muscle endurance. Although other studies have shown that several variables influence IME in the adult population [4, 9], there are still no prediction models for the pediatric age group. Therefore, the aim of the present study was to generate reference values for two protocols (incremental threshold loading and maximal loading) of IME in healthy children and adolescents. In addition, the protocols were compared and their reproducibility was assessed.

## Materials and Methods

This is an observational, cross-sectional study, in children and adolescents from five schools in Porto Alegre, Brazil (4 public and 1 private), from June 2013 to May 2015. The study was approved by the Research Ethics Committee of Pontifícia Universidade Católica do Rio Grande do Sul (19505913.2.0000.5336). All legal guardians of the participants signed the free and informed consent form prior to inclusion. Also, children and adolescents signed an assent form.

### Population

Healthy children and adolescents from four to eighteen years of age were selected. A respiratory disease questionnaire (Thoracic Society e Division of Lung Diseases—ATS-DLD-78-C), previously adapted and validated for the Brazilian population [10], was used. Children and adolescents with a history of prematurity (<37 weeks), low birth weight (<2500 grams), active smoking, prior history of recurrent or active wheezing, heart disease, neuromuscular, scoliosis and thoracic surgery were excluded from the study. Participants who had respiratory infections on the exam date and were unable to perform the proposed tests satisfactorily were also excluded [11].

The sample size was estimated separately for each IME protocol used, based on the data obtained with the first 50 participants. For the incremental threshold loading protocol, considering the values of the higher sustained load, with a significance of 0.05 and a power of 90%, the estimated sample size was approximately 232 children and adolescents. As for the maximal loading protocol, the estimated number of subjects was approximately 172 children and adolescents.

### Experimental Design

Firstly, participants were invited to participate in the study through their school. After completing and returning the respiratory disease questionnaire and the consent form, participants who met the inclusion criteria were selected and called to perform the test at their school of origin. The evaluations were performed during the school period, in both morning and afternoon shifts. Anthropometric measurements (weight and height), inspiratory muscle strength (maximal inspiratory pressure—MIP) and IME were assessed using the incremental or maximal loading protocols as described below.

### Inspiratory Muscle Endurance

#### Incremental threshold loading protocol

**Fig 1:**
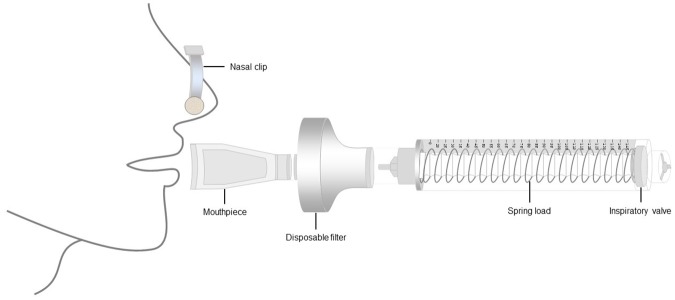
Modified linear workload device.

A linear workload device, Threshold-IMT (Respironics, Andover, USA), was used to determine inspiratory muscle endurance. In order to obtain higher pressures than 41 cmH_2_O, the spring-loaded valve and the device structure were modified. With the assistance of the Biomedical Engineer department at the university, and using the commercial spring of *Threshold*-IMT as a model, a new spring-loaded valve was produced in stainless steel, with an elastic constant of 0.035 N/mm, thereby capable of producing pressures of up to 145 cmH_2_0 (Fig 1).

An incremental threshold loading protocol was used [12], in which participants started inspiring against a fixed load of 30% of MIP during 2 minutes. In order to start the inspiratory flow, it was necessary to generate sufficient inspiratory pressure to open the valve and allow the entrance of air. Every 2 minutes, the load was increased by 10% of MIP. The final result was defined as the highest load, in percentage of MIP, achieved and maintained for at least 1 minute [12]. The breathing pattern was controlled and maintained at 20 breaths/minute by using a metronome. The criteria used to interrupt the protocol were intense fatigue and failure to open the valve at least 3 consecutive times. At the beginning of the protocol and in the last 10 seconds of each load level, participants were requested to rate their perceived dyspnea through the Modified Borg Scale.

#### Maximal loading protocol

The same device described in the incremental threshold loading protocol was used to determine inspiratory muscle endurance through the maximal loading protocol. A protocol adapted from Fiz et al. [3] was used, in which the participants inspired against a fixed load of 70% of MIP. The breathing pattern was also controlled and maintained at 20 breaths/minute by using a metronome. The final result was defined as the time elapsed from the start of the test to exhaustion (Tlim) and expressed as seconds. The criteria used to interrupt the protocol and the assessment of dyspnea were those described above.

#### Reproducibility

To evaluate the reproducibility of both protocols, a subgroup of subjects who performed the incremental threshold loading test and another that executed the maximal loading test were randomly selected to repeat the execution of the protocols. The tests were repeated at least two weeks after the first measurement. Moreover, another subgroup of participants was selected, which performed both protocols of incremental and maximal loading.

### Anthropometric Assessment

Weight and height were measured in triplicate or until two identical values were obtained. Weight was acquired with the participants in an orthostatic position, minimal clothing, without shoes and on a digital scale (G-Tech, Glass 1 FW, Rio de Janeiro, Brazil) previously calibrated with 100-gram precision. Height was acquired from barefooted participants, feet in a parallel position, united ankles, arms extended along the body and head in a neutral position [13]. Height measurements were obtained by a portable stadiometer (AlturaExata, TBW, São Paulo, Brazil) with a precision of 1 mm.

### Inspiratory Muscle Strength

Inspiratory muscle strength assessment was performed by a digital manovacuometer (MVD 300- Globalmed, São Paulo, Brazil) with an amplitude of ± 300 cmH_2_O. MIP was measured with the patient in a sitting position, the torso upright with the hip at a 90° angle and using a nasal clip. A connector between mouth and device was used with a hole to reduce the pressure generated in the oral cavity [14]. Participants were asked to perform maximal inspiratory efforts, from the residual volume and sustained for at least one second, with approximately 1-minute intervals between the measurements [15]. A minimum of three and maximum of nine measurements were used for each test [16]. The test was finalized when technically correct maneuvers were obtained, including three acceptable (without air leak) and two reproducible (variation less than 10% between the two major maneuvers) measurements, wherein the last recorded value could not be higher than the previous one [17]. The highest value obtained in the MIP test, registered in cmH_2_O, was used for the study.

### Statistical Analysis

The Kolmogorov Smirnov test was used to evaluate the main variables of the study, which had a normal distribution. They were thus presented as mean and standard deviation. Categorical data were expressed as total and relative frequency. The Pearson correlation test was used to evaluate the possible correlation of the predictive variables with the respiratory muscle endurance values and also to assess the correlation between the two evaluated protocols.

The association of IME values with the potential predictive values (sex, age, height, weight) was analyzed by a multiple linear regression model. The best variable combination was selected through the *stepwise* method. The test reproducibility was assessed via the calculation of intraclass correlation coefficient (ICC), in which values above 0.75 indicate excellent reproducibility. All analyses and data processing were performed with the SPPSS program version 18.0 (SPSS Inc., EUA). In all cases, differences were considered statistically significant when p<0.05.

## Results

A total of 544 healthy children and adolescents were selected, of which 294 were recruited to perform the IME incremental threshold loading test and 250 to execute the maximal loading protocol. Of those, 20 were not able to perform MIP measurement, 10 did not succeed in the incremental threshold loading protocol and 52 in the maximal loading test. Therefore, the final sample of the present study was composed of 462 participants, of whom 281 performed the incremental threshold loading protocol and 181 the maximal loading test (S1 File). Due to the high failure rate in the 4 and 5-year-old group, the following data included volunteers from 6 to 18 years of age. Characteristics of the sample and inspiratory muscle endurance values classified by sex are presented in S1 Table.

### Incremental Threshold Loading Protocol

A total of 294 participants performed the incremental threshold loading protocol with a success rate of 95.6%. As regards the demographic characteristics of included participants, the mean age was 12.5 years, of which 51.2 were female, with a BMI (z-score) of 0.74. The mean MIP (cmH_2_0) was -111.1±26.0 and the IME (cmH_2_0) was -67.6±24.3. Table 1 presents the characterization data of the studied sample.

**Table 1:** Characteristics of the subjects included.

Variables	Incremental loading protocol (n = 281)	Maximal loading protocol (n = 181)
*Demographics*		
Age (years)	12.5±3.3	12.2±3.7
Sex female (n, %)	141 (50.2)	94 (51.9)
*Anthropometrics*		
Height (cm)	151.2±16.6	148.8±16.8
Weight (kg)	49.3±17.5	46.4±17.2
BMI (total)	20.8±4.2	20.2±4.1
BMI (z-score)	0.74±1.1	0.62±1.1
*Respiratory Muscle Strength*		
MIP (cmH_2_O)	-111.1±26.0	-120.5±23.3
*Inspiratory Muscle Endurance*		
Absolute value (cmH_2_O)	-67.6±24.3	-
% of maximal load	60.3±13.1	-
Time limit (s)	-	807.2±424.7

Data expressed as mean ± standard deviation or as total frequency (relative); BMI: body mass index (weight/height^2^); MIP: maximal inspiratory pressure; s: seconds.

**Fig 2:**
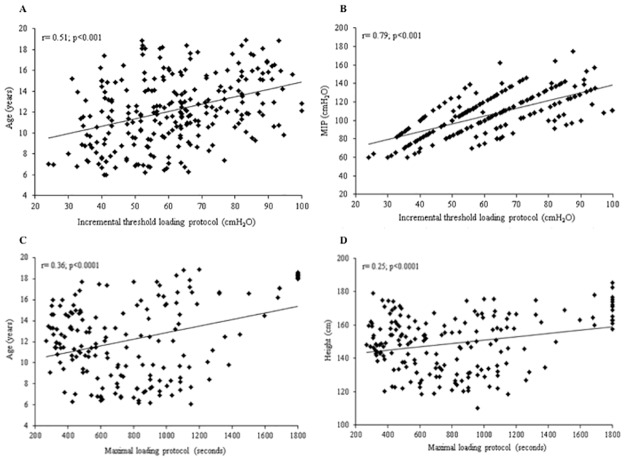
Correlations between IME, evaluated through the incremental threshold loading protocol, and age (A) and MIP (B) and between IME, evaluated through the maximal loading protocol, and age (C) and height (D).

Correlations between IME of the incremental threshold loading protocol and anthropometric characteristics showed moderate and positive results for age (r = 0.51; p = 0.0001), MIP (r = 0.79; p = 0.0001), weight (r = 0.52; p = 0.0001) and height (r = 0.53; p = 0.001) (Fig 2). Subsequently, the stepwise method was used in a multiple linear regression model aiming to obtain the best combination of independent variables to estimate the IME values. MIP and age contributed to explain 66% of the IME in the evaluated sample (Table 2).

**Table 2:** Multiple linear regression models for IME in both protocols.

Protocol	B	SE	Beta	95%CI	R^2^	P
**Incremental loading**						
Constant	-23.861	4.149	-	-	0.657	<0.0001
MIP (cmH_2_O)	0.654	0.036	0.699	0.583 a 0.726	-	<0.0001
Age (years)	1.493	0.280	0.208	0.942 a 2.043	-	<0.0001
**Maximal loading**						
Constant	1195.881	405.652	-	-	0.151	0.004
Age (years)	77.406	18.009	0.657	41.868 a 112.944	-	<0.0001
Height (cm)	-8.998	3.974	-0.356	-1.155 a -16.841	-	0.025

IME: inspiratory muscle endurance; MIP: maximal inspiratory pressure; B: non-standardized coefficient; SE: standard error; CI: confidence interval; R^2^: coefficient of determination.

The following was the best model obtained in multiple regressions to estimate the dependent variable (IME) and it was used to generate the reference equation. As the sex variable did not significantly influence the model, the following formula was obtained to predict the IME values in boys and girls:

$$IME\;\left(cmH_{2}O\right)\;=\;-23.861+\left[MIP\;\left(cmH_{2}O\right)\;\times \;0.654)\right]+\left[\left(Age\;\left(years\right)\;\times \;1.493\right)\right]$$

$$R^{2}=0.657;Standard\;error\;of\;the\;estimate=14.297$$

A strong and positive correlation was demonstrated (r = 0.81; p = 0.0001) (Fig 2) when correlating the values predicted by the proposed equation with the absolute values of IME from participants that performed the incremental threshold loading protocol.

### Maximal Loading Protocol

A total of 250 participants performed the maximal loading protocol with a success rate of 72.4%. The mean age of the participants included was 12.2 years, of whom 51.9% were female, with a BMI (z-score) of 0.62. Mean MIP was 120.5±23.3 and Tlim (s) was 807.2±424.7. Table 1 presents the characteristics of these participants.

Correlations between IME of the maximal loading protocol and anthropometric characteristics demonstrated weak and positive results for age (r = 0.36; p = 0.0001), weight (r = 0.24; p = 0.0010) and height (r = 0.25; p = 0.0010) (Fig 2). In the best model obtained by the multiple linear regression, age and height explained 15% of IME (Table 2). Similarly to the incremental threshold loading protocol, sex did not influence the model. Therefore, the generated model to predict the IME Tlim for boys and girls was the following:

IME (seconds) = 1195.881+[Age (years) × 77.406)]−[(Height (cm) × 8.998)]

$$R^{2}=0.151;Standard\;error\;of\;the\;estimate=393.447$$

**Fig 3:**
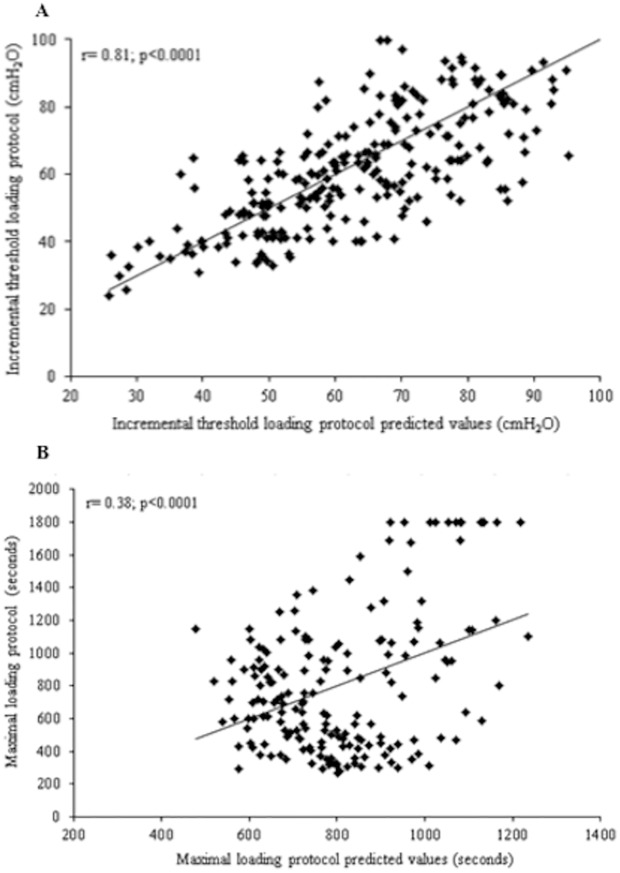
Correlations between the absolute values of IME in the incremental threshold loading (A) and maximal loading (B) protocols with the values predicted by the proposed equations.

A weak and positive correlation (r = 0.38; p = 0.0010) when values of Tlim predicted by the proposed equation were correlated to the absolute values performed by the children and adolescents in the maximal loading protocol was shown in Fig 3.

### Reproducibility

**Fig 4:**
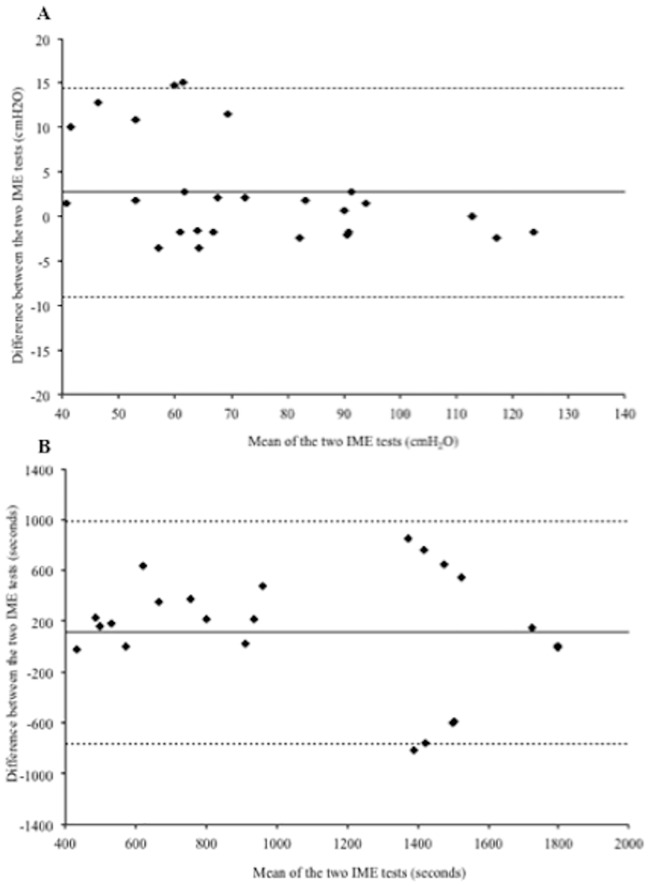
Bland-Altman plots showing the individual differences between both evaluations of the incremental threshold loading (A) and maximal loading (B) protocols, in relation to the mean values of IME. The solid lines indicate the mean differences between the measurements and the dotted lines indicate the 95% limits of agreement.

The reproducibility of tests was evaluated by the ICC. A 26-participant subgroup performed the incremental threshold loading protocol. The mean IME in the first and second evaluation was 72.3+24.0 and 75.0+21.3, respectively, with an ICC of 0.96. As for the second protocol, 28 participants performed the maximal loading test and the mean Tlim was 1162.7+593.3 in the first test and 1272.0+501.3 in the second, with an ICC of 0.69. Fig 4 shows the Bland-Altman plot demonstrating the excellent reproducibility of the incremental threshold loading test and the low ICC of the maximal loading protocol.

Furthermore, 37 participants performed both IME protocols. There were no significant correlations between the protocols when results of the incremental threshold loading protocol were correlated with values obtained in the maximal loading test, either in absolute or in percentage values.

## Discussion

IME measurement can contribute to the evaluation of the respiratory system function in several clinical disorders. However, the lack of reference values for the pediatric age group may hinder the interpretation of findings. The present study presents multiple linear regression models, for two types of protocols (incremental and maximal loading) and the relation between the main independent variables and IME prediction. Although muscle endurance in the incremental threshold loading protocol has demonstrated moderate and positive correlations with age, height, weight and MIP, only age and MIP were included as predictive variables. As for the maximal loading protocol, age, weight and height had weak and positive correlations with muscle endurance. However, the only variables included in the equation were age and height.

To the best of our knowledge, this is the first study that proposed to generate reference values for IME in the pediatric age group. Although several studies [4–6, 9, 18, 19], have evaluated IME in the adult population, only 4 aimed to generate reference values [3, 4, 9, 20]. Among these, the study of Kroff and Terblanche [20] generated reference values for adult athletes, showing a strong correlation between respiratory muscle endurance and physical characteristics, maximal ventilatory ventilation (MVV), forced expiratory volume in the first second (FEV_1_), MIP, maximal expiratory pressure (MEP) and the expiratory peak flow (EPF). When presenting the regression model, only the kinanthropometric variables (sex, relative sitting height) and lung function (FEV_1_, PEF) were included in the equation. In the Johnson et al. study [4], endurance was correlated to age, height and respiratory muscle strength variables. However, only age and sex were included in the prediction model. On the contrary, Fiz et al. [3], reported correlations of IME with FEV_1_, forced vital capacity (FVC), age, height and respiratory muscle strength. In addition, there were negative correlations between BMI and IME (maximal threshold loading protocol) in males. However, age and sex were the only variables that influenced endurance. On the other hand, Reiter et al. [19] found no correlations between endurance and anthropometric variables, MVV and maximal respiratory pressure. This study reported correlations between IME and the BORG scale, but no reference values were generated due to the high variability found. Taken together, the results found for adults mostly corroborate our results, in which the anthropometric variables and respiratory muscle strength were correlated to IME. However, we found no significant influence of sex and spirometric variables were not assessed. The prediction power (coefficient of determination—R^2^) of the equations in these studies [3, 4, 20] varied from 13.3 to 77%, whereas our data demonstrated a power of 65% (incremental threshold loading) and 15% (maximal loading).

Incremental and maximal loading are apparently the most frequently used protocols in the adult population [3–6, 18, 19], although MVV [20] was also described. In the present study, two protocols (incremental and maximal loading) were used and the results indicated that the incremental threshold loading protocol is more reliable, as it shows less variability and a greater power of prediction. Although there are no pediatric studies to compare, previous evidence in adults has also demonstrated a high variability between different age groups [19], a lower sensitivity to detect an intervention effect [21] and a higher coefficient of variation [5] by using Tlim protocols. Present data demonstrated that only 10 participants (4.4%) did not succeed in the incremental threshold loading protocol, as compared to 52 participants (27.6%) in the maximal loading test. We believe that the longer duration of the maximal loading protocol may have contributed to increase the failure rate. It is possible that the lower tolerance to discomfort in children, rather than fatigue, would help to explain these findings, although no similar studies are available for comparison. Therefore, we suggest that IME evaluation for the pediatric population should be performed by using the incremental threshold loading protocol.

The reproducibility for IME in the adult population was assessed only in one [3] of the three studies [3, 4, 20] reporting reference values. Fiz et al. [3] demonstrated a variation of 2% in the incremental loading protocol and of 4% in the maximal loading, but no ICC was shown. Our results demonstrated an excellent reproducibility for the incremental threshold loading protocol (ICC of 0.96) and a moderate reproducibility for the maximal loading test (ICC of 0.69). These data indicate that the IME assessment, both in the adult and in the pediatric population, is apparently more reproducible with the incremental threshold loading protocol. Despite the fact that the same methodology was used in both studies, it is possible that a bigger demonstration and longer measurement time is necessary to perform the test in children. Nevertheless, our data demonstrated that even in a young sample, it is possible to satisfactorily perform maneuvers and generate a quality exam.

The study also presents some limitations. There is a need to adapt the IME device used, since the original equipment has a maximal limit of 41 cmH_2_0. However, different possibilities of adaptation have been described [3, 22–24], considering that IME evaluation requires a high percentage of the inspiratory muscle strength load. The sample size may also constitute a limitation of the study. Although sample and power of analysis were calculated, a larger sample would make results more representative. In addition, data presented here may not be fully reflective of children and adolescents from other ethnic backgrounds.

In conclusion, the results presented here demonstrate that age, height and MIP can explain IME in healthy children and adolescents. Furthermore, the incremental threshold loading protocol presented more reliable results and should be the model of choice to evaluate IME in the pediatric age group. The generation of reference equations can contribute to a better evaluation and follow up of individuals with respiratory muscle dysfunction.

## Supporting Information

S1 Table Characteristics of the sample and inspiratory muscle endurance values classified by sex. Data expressed as mean ± standard deviation; BMI: body mass index (weight/height^2^); MIP: maximal inspiratory pressure; s: seconds.
(DOCX)

S1 File Primary data. Primary data of the study.
(XLSX)
