# Supplementary material for: Reference Values for Inspiratory Muscle Endurance in Healthy Children and Adolescents
Source: PLoS One. 2017 Jan 25;12(1):e0170696. doi: 10.1371/journal.pone.0170696 (PMC5266249; doi:10.1371/journal.pone.0170696)
Supplement: S1 Table — Data expressed as mean ± standard deviation; BMI: body mass index (weight/height2); MIP: maximal inspiratory pressure; s: seconds. (DOCX) [file pone.0170696.s001.docx]

S1 Table**.** Characteristics of the sample and inspiratory muscle endurance values classified by sex.

| **Variables** | **Incremental loading** | | **Maximal loading** | |
| --- | --- | --- | --- | --- |
|  | Boys | Girls | Boys | Girls |
| *Demographics* |  |  |  |  |
| Age (years) | 12.6±3.4 | 12.6±3.4 | 12.3±3.7 | 12.3±3.7 |
| *Anthropometrics* |  |  |  |  |
| Height (cm) | 152.3±18.4 | 150.1±14.7 | 150.1±18.8 | 147.7±14.8 |
| Weight (kg) | 49.6±19.5 | 49.1±15.5 | 46.4±19.1 | 46.5±15.4 |
| BMI (total) | 20.5±4.3 | 21.2±4.2 | 19.8±4.4 | 19.8±4.4 |
| BMI (z-score) | 0.71±1.2 | 0.78±1.1 | 0.54±1.2 | 0.69±1.1 |
| *Respiratory Muscle Strength* |  |  |  |  |
| MIP (cmH_2_O) | -118.0±28.2 | -104.4±21.6 | -125.4±25.8 | 116.1±20.0 |
| *Inspiratory Muscle Endurance* |  |  |  |  |
| Absolute value (cmH_2_O) | -74.5±26.5 | -60.9±19.9 | - |  |
| % of maximal load | 62.6±12.8 | 58.2±13.1 | - |  |
| Time limit (s) | - |  | 825.9±447.4 | 790.1±404.2 |

Data expressed as mean ± standard deviation; BMI: body mass index (weight/height^2^); MIP: maximal inspiratory pressure; s: seconds.
